# Integrated Pest Management techniques in a Kinnow mandarin (*Citrus reticulata* Blanco) orchard with an emphasis on yield improvement

**DOI:** 10.1016/j.heliyon.2025.e42574

**Published:** 2025-02-08

**Authors:** Prabhu Narayan Meena, D. Raghavendra, Satyendra Singh, Narendra Kumar, Mukesh Kumar Khokhar, Subhash Chander, Milan Kumar Lal, Rahul Kumar Tiwari, Ravinder Kumar

**Affiliations:** a ICAR- National Research Centre for Integrated Pest Management, Rajpur Khurd, New Delhi, 110068, India; b Krishi Vigyan Kendra, Sadalpur, Chaudhary Charan Singh Haryana Agricultural University, Hisar, 125052, Haryana, India; c Division of Crop Physiology and Biochemistry, ICAR-National Rice Research Institute, Cuttack, Odisha, India; d Division of Crop Protection, ICAR-Indian Institute of Sugarcane Research, Lucknow, Uttar Pradesh, 226002, India; e Division of Plant Pathology, ICAR-Indian Agricultural Research Institute, New Delhi, 110012, India

**Keywords:** Kinnow mandarin, Psylla, Whitefly, Sooty mould, Dieback, Natural enemies, IPM strategy and economics

## Abstract

Kinnow mandarin (*Citrus reticulata* Blanco) is a valuable fruit crop mainly grown in the North Indian states of India due to its high-quality juice content. Psylla (*Diaphorina citri* Kuwayama), whitefly (*Dialoeurodes citri* Ashmead), sooty mould (*Capnodium citri*) and dieback (*Colletotrichum gloeosporioides*) pests are the most important biotic constraints affecting its fruit yield up to 70 percent. To manage these pests, farmers often use mixture of non-label claim pesticides (quinalphos 25%EC, lambda-cyhalothrin 2.5%EC, diafenthiuron 50%WP, chlorantraniliprole 18.5 %, cymoxanil 8 % + mancozeb 64 % WP, etc.) without achieving the desired effect. Hence, area-wide implementation of the Integrated Pest Management (IPM) strategy in Kinnow mandarin was implemented during 2021–2023 covering 5 villages at Hisar, Haryana. Among the IPM strategy, installing yellow sticky traps @ 20/ha, neem seed kernel extract spray @ 5 %, and imidacloprid 17.8SL @ 0.3 % reduced the whitefly and psylla populations. The spray of 1 % starch and dipping infected fruits in a bleaching solution @ 0.1 % reduced the severity of sooty mould disease. Pruning and destruction of dead twigs followed by a spray of copper oxychloride 50 WP @ 0.3 % were found very effective too. The lowest average population of psylla and whitefly were recorded in T_1_-IPM compared to T_2_-farmer practice and T_3_-control, respectively. Minimum average disease severity of sooty mould and dieback was noticed in T_1_-IPM compared to T_2_-farmer practice while the highest disease severity was recorded in T_3_-control. Population dynamics of psylla, whitefly and sooty mould, dieback severity, and Area Under Diseases Progress Curve (AUDPC) were found to vary during 11th to 52nd standard meteorological week (SMW). They were observed to be highest in T_3_-control treatment, followed by T_2_-farmer practice and T_1_-IPM. The highest natural enemy's populations (Coccinellid, *Chrysoperla,* and spider) were recorded in T_3_-control followed by T_1_-IPM, and then in T_2_-farmer practice treatment. The highest average fruit yield and B: C ratio was recorded in T_1_-IPM compared to T_2_-farmer practice and T_3_-control treatment. The validated IPM strategies can be adopted by Kinnow mandarin growing farmers as an economically viable option for the management of psylla, whitefly, sooty mould, and dieback pests.

## 1 Introduction

Citrus is one of the most relevant fruit crops cultivated in sub-tropical, tropical, and temperate regions across the globe. It has a tremendous social, cultural, and economic influence on our society [1]. The citrus fruits were the second most produced fruit worldwide, and their cultivated area is spread over more than 10.2 million hectares, producing 161.8 million tonnes. China, Brazil, India, Mexico, Spain, USA, and Turkiye are the top citrus producer countries in the world [2]. In Pakistan, especially Punjab, Sargodha, Mianwali, Multan, Toba Tek Singh, Sahiwal, Lahore, Sialkot, and Gujranwala districts produced 98 % of total citrus production. Pakistan cultivated citrus crop on an area of 0.44 million acres with production of 2.18 million tons [3]. It is considered as important tropical fruit crop in India which plays a vital role in the fruit economy of the country, next to mango and banana. India is the home of many citrus fruits, and their cultivated area is spread over more than 109.6 million hectares with a production of 14.25 million tonnes [4]. India ranks fourth among the top citrus-producing countries in the world. In India, citrus is primarily grown in Assam, Andhra Pradesh, Maharashtra, Punjab, Haryana, Karnataka, Uttaranchal, Bihar, Orissa and Gujarat states. Among the citrus fruits, mandarin also known as Kinnow is the most valuable fruit crop that is mostly grown in Haryana, Punjab, and Rajasthan states. The cultivated area under Kinnow mandarin fruit is being stretched from arid and semi-arid regions due to its growing demand in domestic and international consumer markets [1]. The cultivated area under citrus fruits in Haryana is 25.17 thousand hectares with a production of 359.08 thousand million tonnes in which Kinnow mandarin occupied 90 % in both area and production [5]. In Haryana, it is extensively grown in the districts of Hisar, Bhiwani, Sirsa, Mahendergarh and Ambala [6]. Kinnow mandarin, belonging to the family Rutaceae is a hybrid of two citrus, cultivars - “King” (*Citrus nobilis*) x “Willow Leaf'' (*Citrus deliciosa*). This “easy peel” citrus has assumed special economic importance and export demand due to its high juice content, special flavour, and as a rich source of vitamin C [7]. In north India, the cultivation of other mandarins is limited due to the acidity and puffiness of the fruit. Kinnow mandarin has superseded the other oranges in terms of its high yield, good quality fruits, prolific bearing, high juice content, sweetness, and resistance to decline.

The productivity and quality of citrus groups are severely affected by several factors, in which pests being one of them. More than 250 insect pests were reported to attack citrus in India. A total of 22 insect species in Punjab [[8], [9], [10]] 50 in Karnataka, 42 in the north-eastern hill region [11], 20 in West Bengal and about a dozen in central India have been reported on different cultivars. Further, the pattern of pest occurrence and distribution in India indicated that about 21 species are common throughout the citrus growing areas, and the remaining species may occur in some areas either occasionally or rarely. Of the reported pests, Asian citrus psyllid, *Diaphorina citri* (Kuwayama) (Hemiptera: Aphalaridae), whitefly, *Dialoeurodes citri* (Ashmead) (Homoptera: Aleyrodidae), dieback, *Colletotrichum gloeosporioides*, (Melanconiales: Glomerellaceae) and sooty mould, *Capnodium citri*, **(**Capnodiales: Capnodiaceae) are major threats to Kinnow mandarin which cause economic losses from nursery to harvesting [12,13]. Psylla is active on young flush from spring to autumn but more serious damage is done during flowering and fruit-set stage. Nymphs and adults suck the cell sap from the leaves, tender shoots, and flowers and cause curling of leaves, defoliation, and drying of twigs are major symptoms and reduced citrus production up to 95 % [14]. Nymphs secrete honeydew which attracts sooty mould fungus [15]. In case of severe attack, the leaf buds, flower buds, and leaves may wilt and die. Similarly, whitefly nymphs and adults suck the plant sap and secrete honeydew due to which sooty mould develops on the leaves. Severe infestation results in black layer manifestation, covering entire plant parts including fruits. Psylla, whitefly (11–30 %), and other sucking insects cause 70.92 % fruit yield loss in Kinnow mandarin [16].

The sooty mould disease is characterized by the presence of a black velvety thin membrane covering the leaf lamina. In severe cases, the tree completely turns black with mould on the entire surface of twigs and leaves [17] and causes a 10–15 % loss in fruit yield [15]. The fungus multiplies on the honeydew secreted by the insects (psylla and whitefly) and spreads on the plant surface making it black and ugly owing to the masses of black spores on the leaf surface [18]. During flowering times, its attack results in reduced fruit set and premature flowering and fruit drop. The mould deposit may delay in the development of fruit colour [19] and difficult to remove from the fruit surface which reduces the market value and leads to reduced farmers income. Dieback symptoms include the complete decline of trees through the rotting of rootlets, girdling of the trunk, and dropping and blightening of leaves. The disease starts from the apical part of the shoots and under favorable climatic conditions rapidly spreads downwards up to the base of shoots which show signs of wilting and ultimately die [20,21]. Dieback diseases reduce 43.80 % of fruit yield in the orchard [22].

At present, strategies for the management of these pests are mainly focussed on synthetic pesticides which are overloading the food chain and causing human health hazards, chemical residue in the soil, lowering the natural enemy's population, disturbing the natural beneficial microorganisms and resurgence, and resistance problem in pests [23]. Most of the farmers follow management practices based on advice of local pesticide dealers which increase the cost of plant protection and pollute the natural ecosystem. Moreover, farmers are following the calendar-based pesticide spray against Kinnow mandarin pests. Therefore, it is necessary to reduce the pesticide load from the food chain which could be achieved suitably by developing and implementing the IPM strategies. Besides, the alternative option of pesticides might be an incorporation of biopesticides [[24], [25], [26], [27]] that play a pivotal role as a component of IPM strategy. To overcome these problems and to achieve our goals, it is essential to put all IPM strategies/options available holistically and practiced in farmers participatory mode to evolve a comprehensive IPM technology for the better management of pests. These Integrated Pest Management (IPM) strategies prevent pest resurgence, maintain natural enemy diversity, reduce pesticide residues, and increase the economic yield of the crop. Hence, the present investigation was carried out for three years against major Kinnow mandarin pests to assess and devise pest management techniques that can be adopted widely as a component of Integrated Pest Management.

## 2 Materials and methods

### 2.1 Description of the study area

The experiment was implemented in a farmers' participatory mode in five villages (Kisangarh 24.21°N and 74.51°E; Daroli 29.27°N and 75.39°E; Khara Barwala, 29.38°N 75.92°E; Sadalpur 29.19°N and 75.27°E and Chuli 29.27°N and 75.47°E) from 2021 to 2023 in collaboration and supervision of Krishi Vigyan Kendra (KVK), Sadalpur, Choudhary Charan Singh Haryana Agricultural University, Hisar (Haryana).

#### 2.1.1 Experimental design, IPM strategies, and field management

The IPM practices were validated against psylla, whitefly, dieback, and sooty mould pests following Randomized Complete Block Design (RCBD) having plant spacing 6 × 6 m in 1-acre plot size. A total of twenty-five plants in five replications from each orchard were used for recording the pests observations. A total of 120 plants/acre of test variety (local) were maintained in the orchard. The average age of Kinnow plants in all orchards was in the range of 8–10 years. A total of twenty orchards of T_1_-IPM and T_2_-farmer practices were selected from five villages. Besides, control plants were also maintained in the orchard for comparison with T_1_-IPM and T_2_-farmer practices at each location. Different Integrated Pest Management (IPM) strategies mentioned in Table 1 *viz*; deep summer ploughing; pruning the excess branches and bottom leaves of the tree for proper ventilation; destruction of rotten and dropped infected fruits; regular monitoring of orchard to assess the damage and severity of pests; Installation of yellow sticky traps @ 20/ha followed by a need-based spray of neem seed kernel extract (NSKE) @ 5 % on young leaves and if insect populations exceeded above ETL then applied need-based spray of label claim imidacloprid 17.8SL @ 200 ml in 500 L of water or thiamethoxam 25 % WG in 50g/500 L water to manage psylla and whitefly; spray of 1 % starch against sooty mould disease during August, September and October months and dipping sooty mould infected fruits in a bleaching solution @ 0.1 %; pruning and destruction of dieback infected dead twigs in the month of March and July followed by spray of copper oxychloride 50 WP @ 3 g/l were validated in T_1_-IPM treatment. In T_2_-farmer practice treatment, farmers used only FYM (100 kg) and urea (700g/plant) and not practiced IPM techniques. Injudiciously they used 8–12 sprays of pesticides such as copper oxychloride 50 WP @ 3 g/l (dieback); cymoxanil 8 % + mancozeb 64 % WP @ 1.5 g/l (sooty mould); carbendazim 50 WP @ 2 g/l (dieback); imidacloprid 17.8SL @ 2 ml/l (whitefly); ethion 50 EC @ 1 ml/l (whitefly); beta-Cyfluthrin + imidacloprid 300 OD (8.49 + 19.81 % w/w) @ 1 ml/l (psylla and whitefly); lambda cyhalothrin 2.5 % EC @ 1.5 ml/l (psylla); chlorpyrifos 20 % EC @ 2 ml/l (psylla); diafenthiuron 50 % WP @ 1 g/l; quinalphos 25 % EC@ 2 ml/l and chlorantraniliprole 18.5 % w/w@ 1.5 ml/l (psylla and whitefly); farmers mostly depend on local pesticide dealer for advice and used higher than the recommended dose of pesticides. In T_3_-control treatment only water spray was undertaken.

**Table 1:** Details of practices used in IPM, farmer practice and control.

Management practice	Details
T_1_- Integrated pest management (IPM)	**Common practices:** Deep summer ploughing; pruning the excess branches and bottom leaves of tree for proper ventilation; destruction of rotten and dropped infected fruits; regular monitoring of orchard to assess the damage and severity of pests; **IPM practices:** 1 Installation of yellow sticky traps @ 20/ha followed by need based spray of neem seed kernel extract (NSKE) 5 % on young leaves and if populations of insect is above ETL then use need-based spray of label claim imidacloprid 17.8SL @ 200 ml in 500 L of water or thiamethoxam 25 % WG in 50 gm/500 L water to manage psylla and whitefly. 2 Spray of 1 % starch against sooty mould disease during August, September and October months and dipping sooty mould infected fruits in a bleaching solution. 3 Pruning and destruction of dieback infected dead twigs in the month of March and July followed by spray of copper oxychloride @ 3 g/l. 4 Spraying of aureofungin/bavistin +2,4-D @ 0.4g/10 g + 0.1g/10/l water to reduce fruit drop and use of gibberellic acid when cotton or other broad leaves crop is cultivated in or around the orchard. 5 Conservation of natural enemies (Spider, Chrysoperla and Coccinellidae) and enhance their population through ecological engineering (marigold, sunflower etc.) and growing intercrop with onion/garlic/chickpea between rows of trees which helps in moisture and natural enemies' conservation and reduce irrigation frequency, and suppression of weeds.
T_2_- Farmer practice (FP)	1 Generally farmers not practiced IPM techniques and injudiciously used 8–12 sprays of pesticides such as copper oxychloride 50 WP @ 3 g/l (dieback); cymoxanil 8 % + mancozeb 64 % WP @ 1.5 g/l (sooty mould); carbendazim 50 WP @ 2 g/l (dieback); imidacloprid 17.8SL @ 2 ml/l (whitefly); ethion 50 EC @ 1 ml/l (whitefly); beta-Cyfluthrin + imidacloprid 300 OD (8.49 + 19.81 % w/w) @1 ml/l (psylla and whitefly); lambda cyhalothrin 2.5 % EC @ 1.5 ml/l (psylla); chlorpyrifos 20 % EC @2 ml/l (psylla); diafenthiuron 50 % WP @1 g/l; quinalphos 25 % EC@2 ml/l and chlorantraniliprole 18.5 % w/w@ 1.5 ml/l(psylla and whitefly). 2 Farmers mostly depend on local shop dealer for advice and used higher than recommended dose of pesticides.
T_3_- Control	Control without using of chemical and non-chemical spray.

The recommended dose of farm yard manure (FYM) (80 kg), urea (500g/plant), single super phosphate (2 kg/plant) and muriate of potash (1.75kg/plant) minerals like manganese, copper, sulphur, boron, zinc were applied as per age and required stage of plant in T_1_-IPM. In T_2_-farmers practice, only FYM (100 kg) and urea (700g/plant) were used. Similarly, in T_3_-control plants, only FYM (80 kg) was applied. To improve fruit size and increase yield in Kinnow mandarin, three foliar sprays of 1.0 % potassium nitrate (10 g/L) were given at the end of May, June and July in T_1_-IPM. The drip irrigation method was followed by farmers in T_1_-IPM, T_2_-farmer practice, and T_3_-control treatments. Yellow sticky traps (3 × 5 inches) were purchased from Pest Control of India Pvt Ltd. (PCI), Mumbai, India, while yellow karina f-1 hybrid marigold seeds (Sagar Biotech Private Limited) and, sunflower seeds (CCHAU, Hisar) provided by Krishi Vigyan Kendra (KVK), Sadalpur, Hisar which were sown in 5:1 ratio (five rows of Kinnow and one row of marigold) while chickpea was grown in each row. Garlic and onion seedlings were managed by respective orchard farmers. All other IPM inputs like bioagents (produced from NRIIPM, New Delhi), 2,4-D (Kalyani Industries Ltd), copper oxychloride (Rallis India Ltd), NSKE 5 % (Bioprex Labs, Pune), imidacloprid 17.8SL (Bayer India) and thiamethoxam 25 % WG (Syngenta India) etc. were used in IPM orchards.

### 2.2 Identification, monitoring, economic threshold level, and insect pests and disease assessment

Identification of psylla, whitefly, sooty mould, and dieback: The psylla and whitefly were collected and preserved in 70 % ethanol and identified according to taxonomic characteristics mentioned by Refs. [[28], [29], [30]]. *Colletotrichum gloeosporioides*, and *Capnodium citri* pathogens were identified based on morphological and cultural characteristics. The sooty mould black material collected from infected leaves were placed on potato dextrose agar (PDA) medium amended with streptomycin (100 ppm) and incubated at 28 ± 2 °C in BOD for 7 days and identified according to morphological characters (black mycelium and brown colour of spores) given by Refs. [31,32]. Similarly, *Colletotrichum gloeosporioides* infected leaves and twigs were collected from orchards and placed on potato dextrose agar (PDA) medium and incubated at 28 ± 2 °C in BOD for 7 days. Identification was carried out according to morphological characters (fluffy mycelium, initially white gray and produced slightly curved shaped conidia) given by Ref. [33].

Monitoring and observations of pests and natural enemies (spider, coccinellids’ and green lacewing) were recorded on five randomly selected plants each in five replications from four directions of each plant (north, south, east, and west) as per standard metrological week (SMW). The population dynamic of psylla was recorded as a number of nymphs and adults present per 10 cm shoot while, whitefly was recorded as the number of adult whitefly per leaf. The economic threshold level (ETL) for psylla was 6 psylla/shoot while it was 4–10 nymph and adults per leaf for whitefly. Population of spider (adults and spiderlings/m^2^), coccinellids (adults/m^2^) and chrysopids (adults/m^2^) were also recorded [34,35]. Observations on sooty mould disease severity were recorded on infected leaf by using a standard disease rating scale 0–4 [36], where 0 = leaf was free from infection, 1 = 1–10 % of leaf area covered, 2 = 11–25 % of leaf area covered, 3 = 26–50 % of leaf area covered and 4= > 50 % of leaf area covered. Disease severity of dieback was noticed on twigs and branches, recorded as per a disease rating scale of 0–4 [37], where 0 = no sign or symptoms, 1 = 0–25 % affected branches, 2 = 26–50 % affected branches, 3 = 51–75 % affected branches and 4 = 76–100 % affected branches. The ETL of sooty mould and dieback was 10 % (disease score 1) severity on the plant. Disease grades were converted into percentage severity index (PSI) as suggested earlier [38]. Disease incidence (DI) and percent severity index (PSI) were calculated by using the following formulae; Disease incidence (DI) = [Number of infected plants/Total number of plants] × 100; Percent severity index (PSI) = [Sum of numerical ratings/Total number of observations × Maximum disease rating grade] × 100 [39]. Economic parameters like chemical spray (nos), cost of plant protection, cost of cultivation, fruit yield, gross income, net income, net profit, and benefit-cost ratio were (Including cost incurred on labour charges, fertilizer cost, plant protection cost, and income received from fruit yield) calculated considering all inputs used and outputs obtained using the following formula; B: C ratio = [Income received (treatment wise)/cost incurred (treatment wise)] [40].

### 2.3 Area Under Disease Progress Curve (AUDPC)

The AUDPC was calculated from the severity of the disease [41]. For the AUDPC, data’ recorded on different days for dieback and sooty mould disease incidence were used as per the formula:

$$\text{AUDPC}=\Sigma_{i}^{n-1}\;\left(y_{\left(I+1\right)}+y_{i}\;/2\right)\;\left(t_{(1+I}-t_{i}\right)\text{,}$$

Where n, is the number of assessment times; y, disease incidence and t = time (30 days).

### 2.4 Assessment of fruit yield and fruit yield loss

Fruit yield was calculated after harvesting of Kinnow mandarin fruits. In observation of the weight of fresh fruit yield, all fruits of five plants of kinnow mandarin were pooled after being harvested, then mean weights of fruit yield in T_1_-IPM, T_2_-farmer practice and T_3_-control were calculated. Fruit yield loss (FYL) was calculated using the [42] formula: Fruit yield loss (%) = Fruit yield from IPM – Fruit yield from Control/Fruit yield from IPM × 100.

### 2.5 Statistical analysis

Observation recorded on disease and insects were transformed into angular and square root values to normalized data prior to statistical analysis. The data is expressed as the mean ± standard error of mean. Randomized complete block design (RCBD) was used to decrease experimental error. A significant difference (p < 0.05) by Duncan's multiple range test (DMRT) was determined between the treatments. Statistical analysis accomplished by IBM SPSS v 16, Chicago, USA.

## 3 Results

The effectiveness of IPM strategy on the severity of pests and yield in IPM orchards was systemically recorded as per the standard sampling procedures. The economics of IPM strategy were also been worked out in T_1_-IPM, T_2_-farmer practice, and T_3_-control.

### 3.1 Effect of IPM strategy on psylla

**Fig. 1:**
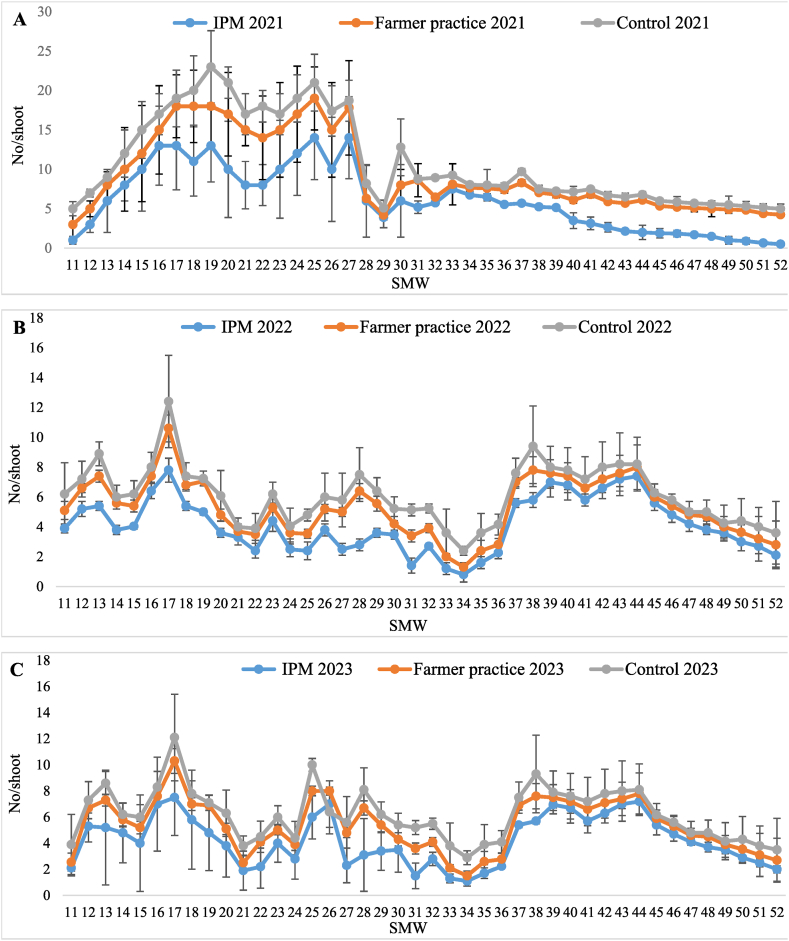
Seasonal population dynamics of psylla in (A) 2021, (B) 2022, and (C) 2023, in Kinnow mandarin; Error bar represent standard errors.

The data on populations of psylla in T_1_-IPM, T_2_-farmer practice, and T_3_-control treatment recorded from 11th −52nd standard metrological weeks (SMW) and are presented in Table 2. It was evident that lowest population of psylla (6.11 ± 0.63, 4.14 ± 0.28 and 4.21 ± 0.29 nymphs and adults/10 cm shoot) occurred in T_1_-IPM compared to T_2_-farmer practice (9.35 ± 0.77, 5.29 ± 0.30 and 5.37 ± 0.32 nymphs and adults/10 cm shoot) and T_3_-control (10.86 ± 0.88, 6.10 ± 0.30 and 6.19 ± 0.31 nymphs and adults/10 cm shoot) during 2021, 2022 and 2023, respectively. From the pooled mean data, it is apparent that the mean population of psylla was below the economic threshold level (6 psylla/shoot) in T_1_-IPM (4.82 ± 0.28 nymphs and adults/10 cm shoot) whereas it exceeded the ETL in T_2_-farmer practice (6.67 ± 0.36 nymphs and adults/10 cm shoot) and in T_3_-control (7.71 ± 0.38 nymphs and adults/10 cm shoot). Highest reduction in psylla population over the control was also observed in T_1_-IPM (37.49 %) compared to T_2_-farmer practice (13.48 %). The psylla population was less in T_1_-IPM compared to T_2_-farmer practice and T_3_-control during the three consecutive years. The psylla appeared during 11th SMW in T_1_-IPM (2.33 ± 0.63/shoot) T_2_-farmer practice (3.55 ± 1.10/shoot) and T_3_-control (5.03 ± 1.77/shoot) and reached peak in 17th SMW (9.43 ± 1.43, 12.97 ± 1.55 and 14.50 ± 2.12/shoot), thereafter its population declined (Fig. 1A–C).

**Table 2:** Effect of IPM strategy on population dynamics of psylla in IPM, farmer practice and untreated control.

SMW	Treatment	2021	2022	2023	Pooled^b^ Mean	Reduction of psylla over Untreated control (%)
		Psylla (No. of nymphs and adults/10 cm shoot)	Psylla (No. of nymphs and adults/10 cm shoot)	Psylla (No. of nymphs and adults/10 cm shoot)		
11–52	T_1_-IPM	6.11 ± 0.63 (2.55)^c^^a^®	4.14 ± 0.28 (2.22)^c^	4.21 ± 0.29 (2.24)^c^	4.82 ± 0.28 (2.38)^c^	37.49
11–52	T_2_-FP	9.35 ± 0.77 (3.14)^b^	5.29 ± 0.30 (2.48)^b^	5.37 ± 0.32 (2.49)^b^	6.67 ± 0.36 (2.73)^b^	13.48
11–52	T_3_-UC	10.86 ± 0.88 (3.36)^a^	6.10 ± 0.30 (2.64)^a^	6.19 ± 0.31 (2.67)^a^	7.71 ± 0.38 (2.93)^a^	–

a Figure in brackets represents the square root transformed value.

b Mean of five replications; Values presented here are the mean ± standard error of the mean; ® means followed by the same superscripts in a column are not significantly different at (P < 0.05) according to DMRT; SMW-Standard Metrological week; T_1_-IPM -Integrated pest management; T_2_-FP - Farmers practice; T_3_-UC -Untreated control.

### 3.2 Effect of IPM strategy on whitefly

**Fig. 2:**
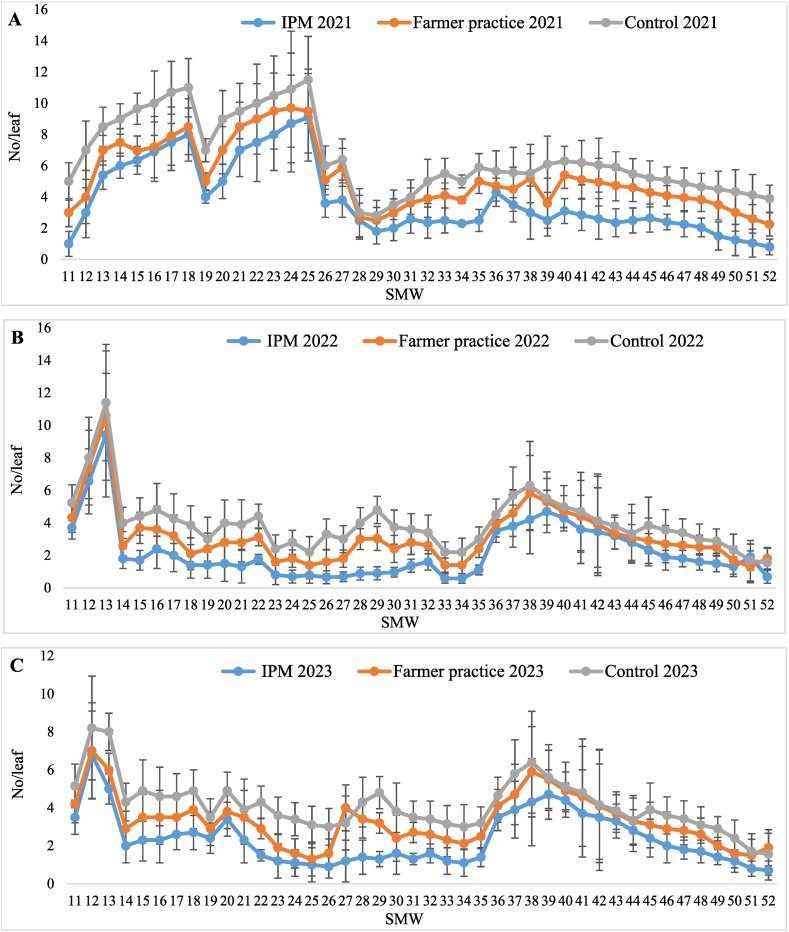
Seasonal population dynamics of whitefly in (A) 2021, (B) 2022, and (C) 2023, in Kinnow mandarin; Error bar represent standard errors.

The data presented in Table 3 revealed that the infestation of whitefly started from 11th SMW and continued until the 52nd SMW. The lowest population of whitefly (3.81 ± 0.36, 2.22 ± 0.25 and 2.36 ± 0.21 nymphs and adults/leaf) was noticed in T_1_-IPM compared to T_2_-farmer practice (5.24 ± 0.33, 3.17 ± 0.27 and 3.26 ± 0.20 nymphs and adults/leaf) and T_3_-control (6.57 ± 0.38, 3.98 ± 0.29 and 4.12 ± 0.21 nymphs and adults/leaf), during three years, respectively. From the pooled mean it is evident that whitefly population was below ETL in T_1_-IPM (2.80 ± 0.19 nymphs and adults/leaf) followed by T_2_-farmer practice (3.89 ± 0.20 nymphs and adults/leaf) while it was above ETL in T_3_-control (4.90 ± 0.22 nymphs and adults/leaf). The highest reduction of the whitefly population (42.86 %) was observed in T_1_-IPM followed by T_2_-farmer practice (20.61 %). Whitefly made its first appearance during 11th SMW with population of 2.73 ± 0.8, 3.84 ± 0.93 and 5.13 ± 1.15/leaf in T_1_-IPM, T_2_-farmer practice and T_3_-control and reached on peak in 13th SMW (6.60 ± 1.83, 7.87 ± 1.93 and 9.30 ± 1.95/leaf) during three years, respectively, thereafter it declined until 35th SMW but again increased from 36th SMW to 41st SMW (Fig. 2A–C).

**Table 3:** Effect of IPM strategy on population dynamics of whitefly in IPM, farmer practice and untreated control.

SMW	Treatment	2021	2022	2023	Pooled^b^ Mean	Reduction of whitefly over Untreated control (%)
		Whitefly (No. of nymphs and adults/leaf)	Whitefly (No. of nymphs and adults/leaf)	Whitefly (No. of nymphs and adults/leaf)		
11–52	T_1_-IPM	3.81 ± 0.36 (2.13)^c^^a^®	2.22 ± 0.25 (1.74)^c^	2.36 ± 0.21 (1.80)^c^	2.80 ± 0.19 (1.92)^c^	42.86
11–52	T_2_-FP	5.24 ± 0.33 (2.46)^b^	3.17 ± 0.27 (2.00)^b^	3.26 ± 0.20 (2.05)^b^	3.89 ± 0.20 (2.20)^b^	20.61
11–52	T_3_-UC	6.57 ± 0.38 (2.71)^a^	3.98 ± 0.29 (2.20)^a^	4.12 ± 0.21 (2.24)^a^	4.90 ± 0.22 (2.41)^a^	–

a Figure in brackets represents the square root transformed value.

b Mean of five replications; Values presented here are the mean ± standard error of the mean; ® means followed by the same superscripts in a column are not significantly different at (P < 0.05) according to DMRT; SMW-Standard Metrological week; T_1_-IPM -Integrated pest management; T_2_-FP - Farmers practice; T_3_-UC -Untreated control.

#### 3.2.1 Effect of IPM strategy on sooty mould

**Fig. 3:**
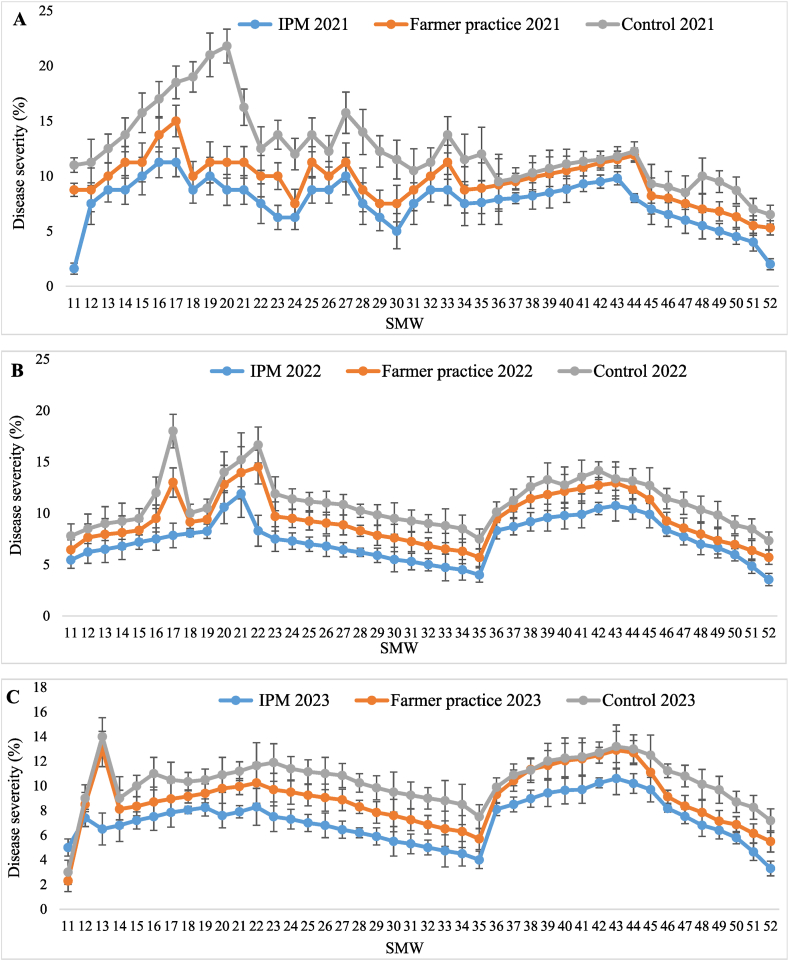
Seasonal disease severity of sooty mould in (A) 2021, (B) 2022, and (C) 2023, in Kinnow mandarin; Error bar represent standard errors.

The highest disease severity of sooty mould was recorded in T_3_-control (12.41 ± 0.53, 11.04 ± 0.37 and 10.39 ± 0.30 %) followed by T_2_-farmer practice (9.60 ± 0.31, 9.35 ± 0.37 and 8.96 ± 0.35 %) and in T_1_-IPM (7.62 ± 0.33, 7.42 ± 0.31and 7.20 ± 0.28 %) during three years, respectively. The pooled mean disease severity of sooty mould was below ETL (score 1) in T_1_-IPM (7.41 ± 0.26 %) and T_2_-farmer practice (9.31 ± 0.30 %) compared to T_3_-control (11.28 ± 0.30 %) treatment. Likewise, the highest reduction in sooty mould severity was also recorded in T_1_-IPM (34.30 %) in comparison to T_2_-farmer practice (17.46 %). Besides, disease incidence of sooty mould was also recorded at 30, 60, 120, 180, 240, and 300 days. The lowest pooled mean incidence (25.48 %) was observed in T_1_-IPM compared to T_2_-farmer practice (55.00 %) and T_3_-control (67.17 %) treatment. As well, highest reduction of sooty mould incidence over control was also seen in T_1_-IPM (62.06 %) compared to T_2_-farmer practice (18.12 %) (Table 4). Lowest sooty mould severity was noticed in 11th SMW (4.02 ± 0.67, 5.83 ± 0.82 and 7.27 ± 0.94 %) in T_1_-IPM, T_2_-farmer practice and T_3_-control thereafter, it increased and reached peak in 43rd SMW (10.38 ± 1.03, 12.45 ± 1.27 and 12.78 ± 1.16 %) (Fig. 3A–C).

**Table 4:** Effect of IPM strategy against sooty mould disease in IPM, farmer practice and untreated control.

SMW	Treatment	2021	2022	2023	2021	2022	2023	Pooled^b^ Mean Severity (%)	Pooled^b^ Mean Incidence (%)	Reduction of sooty mould severity over control (%)	Reduction of sooty mould incidence over control (%)
		Sooty mould severity (%)	Sooty mould severity (%)	Sooty mould severity (%)	Sooty mould incidence (%)	Sooty mould incidence (%)	Sooty mould incidence (%)				
11–52	T_1_-IPM	7.62 ± 0.33 (15.83)^c^^a^®	7.42 ± 0.31 (15.66)^c^	7.20 ± 0.28 (15.43)^c^	28.30 (32.14)^c^	25.33 (30.22)^c^	22.83 (28.54)^c^	7.41 ± 0.26 (15.68)^c^	25.48 (30.32)^c^	34.30	62.06
11–52	T_2_-FP	9.60 ± 0.31 (17.95)^b^	9.35 ± 0.37 (17.67)^b^	8.96 ± 0.35 (17.27)^b^	58.17 (49.70)^b^	54.50 (47.58)^b^	52.33 (46.34)^b^	9.31 ± 0.30 (17.67)^b^	55.00 (47.87)^b^	17.46	18.12
11–52	T_3_-UC	12.41 ± 0.53 (20.46)^a^	11.04 ± 0.37 (19.29)^a^	10.39 ± 0.30 (18.70)^a^	70.67 (57.21)^a^	66.67 (54.74)^a^	64.17 (53.23)^a^	11.28 ± 0.30 (19.55)^a^	67.17 (55.O4)^a^	–	–

a Figures in parentheses are angular transformed values and others are actual disease incidence & severity (DI & PSI); Values presented here are the mean ± standard error of the mean.

b Mean of five replications; ® means followed by the same superscripts in a column are not significantly different at (P < 0.05) according to DMRT; SMW-Standard Metrological week; T_1_-IPM -Integrated pest management; T_2_-FP - Farmers practice; T_3_-UC -Untreated control.

### 3.3 Effect of IPM strategy on dieback

**Fig. 4:**
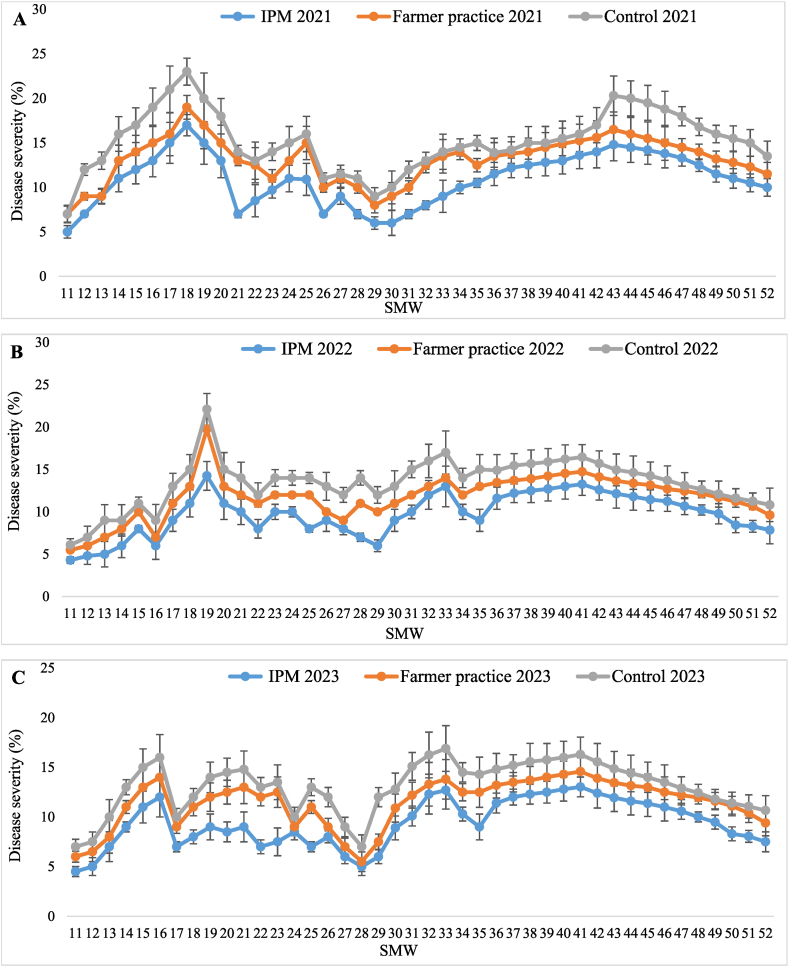
Seasonal disease severity of dieback in (A) 2021, (B) 2022, and (C) 2023, in Kinnow mandarin; Error bar represent standard errors.

Minimum dieback disease severity was recorded in T_1_-IPM (10.94 ± 0.45, 9.72 ± 0.39 and 9.39 ± 0.37 %) while higher severity was observed in T_2_-farmer practice (13.14 ± 0.40, 11.75 ± 0.41 and 11.44 ± 0.37 %) and T_3_-control (15.21 ± 0.52, 13.44 ± 0.44 and 13.08 ± 0.40 %) treatment, during three years, respectively. The least pooled mean severity was noticed in T_1_-IPM (10.01 ± 0.35 %) while the highest severity recorded in T_2_-farmer practice (12.11 ± 0.34 %) followed by T_3_-control (13.91 ± 0.36 %). In similar way, the highest reduction of dieback severity over control was recorded in T_1_-IPM (28.03 %) compared to T_2_-farmer practice (12.94 %). Dieback disease incidence was recorded at 30, 60,120,180, 240, and 300 days in T_1_-IPM, T_2_-farmer practice, and T_3_-control and maximum pooled mean disease incidence (51.72 %) observed in T_3_-control followed by T_2_-farmer practice (33.50 %) and T_1_-IPM (22.00 %). The highest reduction of disease incidence over control was observed in T_1_-IPM (57.46 %) compared to T_2_-farmer practice (35.22 %) (Table 5). The dieback severity appeared in 11th SMW (4.60 ± 0.53, 6.17 ± 0.66 and 6.70 ± 0.83 %) in T_1_-IPM, T_2_-farmer practice and T_3_-control thereafter, it increased and reached peak in 19th (12.75 ± 1.27, 16.25 ± 1.69 and 18.70 ± 1.86 %) and 41st SMW (13.29 ± 1.33, 14.85 ± 1.41 and 16.25 ± 1.69 %) then decreased until 52nd SMW (Fig. 4A–C).

**Table 5:** Effect of IPM strategy against dieback disease in IPM, farmer practice and untreated control.

SMW	Treatment	2021	2022	2023	2021	2022	2023	Pooled^b^ Mean Severity (%)	Pooled^b^ Mean Incidence (%)	Reduction of dieback severity over control (%)	Reduction of dieback incidence over control (%)
		Dieback severity (%)	Dieback severity (%)	Dieback severity (%)	Dieback incidence (%)	Dieback incidence (%)	Dieback incidence (%)				
11–52	T_1_-IPM	10.94 ± 0.45 (19.13)^c^^a^®	9.72 ± 0.39 (18.00)^c^	9.39 ± 0.37 (17.70)^c^	25.17 (30.11)^c^	21.67 (27.74)^c^	19.17 (25.97)^c^	10.01 ± 0.35 (18.33)^c^	22.00 (27.97)^c^	28.03	57.46
11–52	T_2_-FP	13.14 ± 0.40 (21.15)^b^	11.75 ± 0.41 (19.92)^b^	11.44 ± 0.37 (19.65)^b^	36.83 (37.36)^b^	33.33 (35.26)^b^	30.33 (33.42)^b^	12.11 ± 0.34 (20.27)^b^	33.50 (35.37)^b^	12.94	35.22
11–52	T_3_-UC	15.21 ± 0.52 (22.82)^a^	13.44 ± 0.44 (21.39)^a^	13.08 ± 0.40 (21.09)^a^	55.50 (48.16)^a^	51.67 (45.96)^a^	48.00 (43.85)^a^	13.91 ± 0.36 (21.82)^a^	51.72 (45.99)^a^	–	–

a Figures in parentheses are angular transformed values and others are actual disease incidence & severity (DI & PSI); Values presented here are the mean ± standard error of the mean.

b Mean of five replications; ® means followed by the same superscripts in a column are not significantly different at (P < 0.05) according to DMRT; SMW-Standard Metrological week; T_1_-IPM -Integrated pest management; T_2_-FP - Farmers practice; T_3_-UC -Untreated control.

### 3.4 Population dynamic of natural enemies

**Fig. 5:**
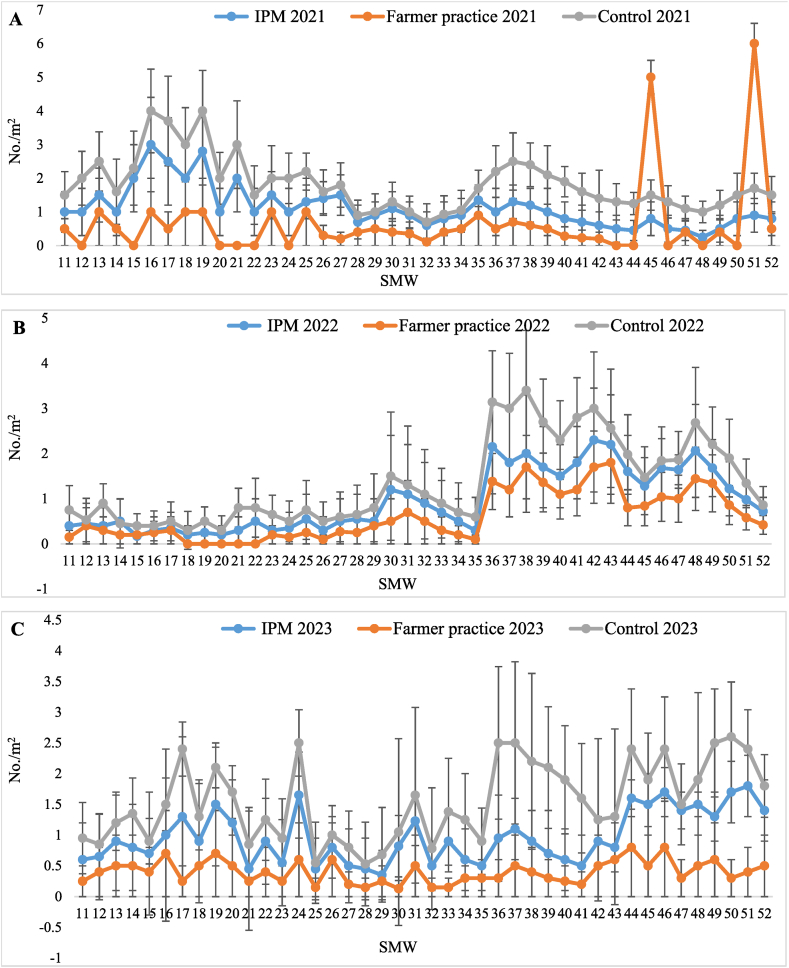
Seasonal population dynamics of natural enemies coccinellid in (A) 2021, (B) 2022, and (C) 2023, in Kinnow mandarin; Error bar represent standard errors.

**Fig. 6:**
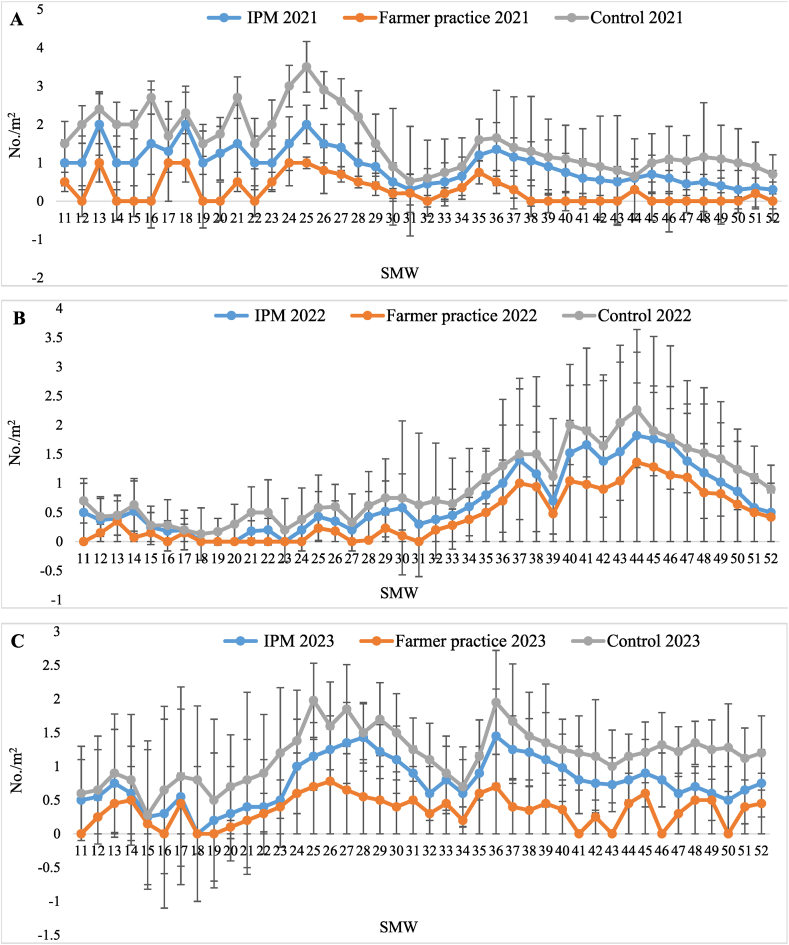
Seasonal population dynamics of natural enemies chrysopid in (A) 2021, (B) 2022, and (C) 2023, in Kinnow mandarin; Error bar represent standard errors.

**Fig. 7:**
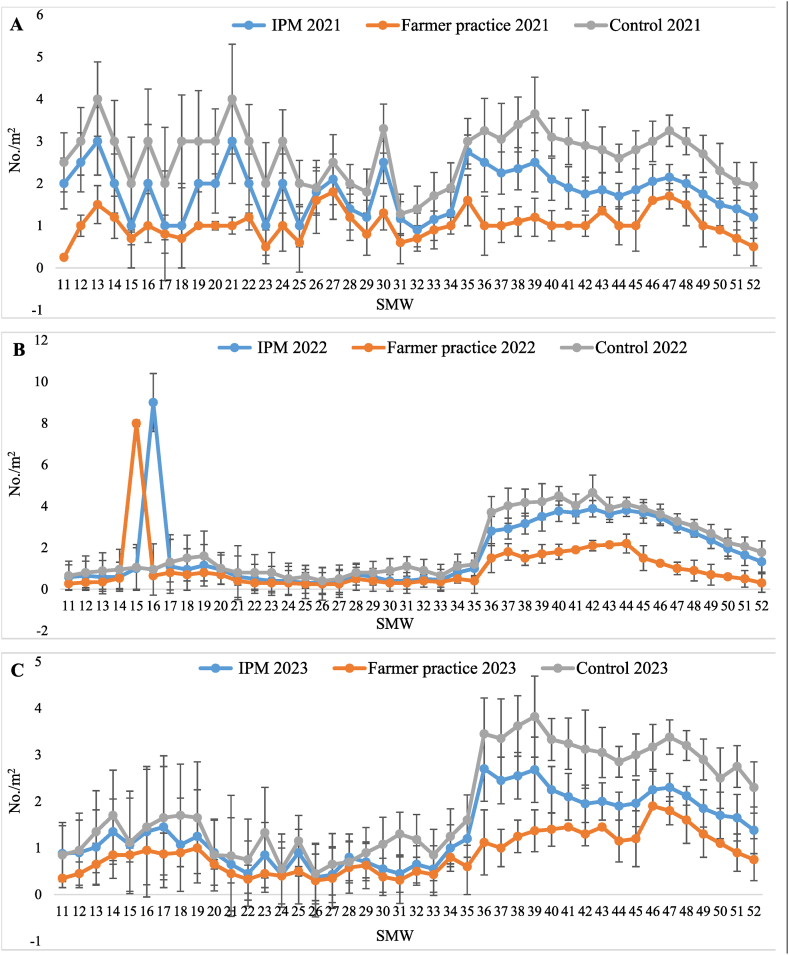
Seasonal population dynamics of natural enemies' spider in (A) 2021, (B) 2022, and (C) 2023, in Kinnow mandarin; Error bar represent standard errors.

Population dynamics of natural enemies (coccinellids, chrysoperla and spider) were recorded from 11th SMW to 52nd SMW in T_1_-IPM, T_2_-farmer practice, and T_3_-control treatments. The population of natural enemies was higher in T_1_-IPM compared to T_2_-farmer practice. The three years pooled average highest populations of coccinellid (1.57/m^2^), *Chrysoperla* (1.21 m^2^) and spider (2.19 m^2^) were recorded in T_3_-control followed by (1.02, 0.80 and 1.60/m^2^) in T_1_-IPM and (0.47, 0.36 and 0.91/m^2^) in T_2_-farmer practice. The first appearance of coccinellid populations dynamic was noticed in the month of March (11th SMW) and it persisted until 52nd SMW in the range of 0.69–2.67/m^2^ in T_3_-control, 0.67 to 1.52/m^2^ in T_1_-IPM and 0.08 to 0.90/m^2^ in T_2_-farmer practice (Fig. 5A–C). Likewise, *Chrysoperla* was observed in the range of 0.82–1.63/m^2^ in T_3_-control, 0.40 to 1.27/m^2^ in T_1_-IPM and 0.00 to 0.70/m^2^ in T_2_-farmer practice (Fig. 6A–C). Population dynamics of spider was also recorded from 11th SMW to 52nd SMW in the range of 0.92–3.90/m^2^ in T_3_-control followed by 0.67–2.89/m^2^ in T_1_-IPM and 0.29 to 1.65/m^2^ in T_2_-farmer practice, respectively (Fig. 7A–C).

### 3.5 Effect of IPM strategy on Area Under Disease Progress Curve (AUDPC)

**Fig. 8:**
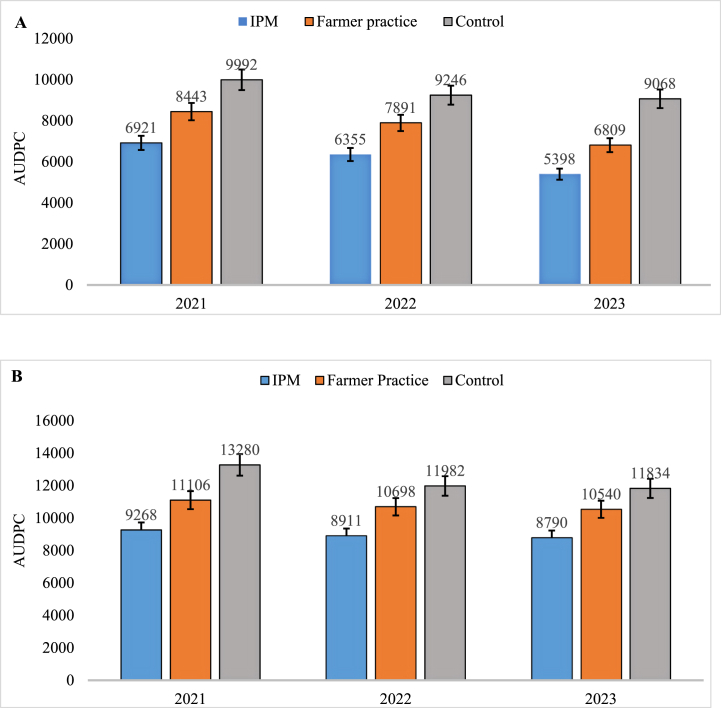
Area Under Diseases Progress Curve (AUDPC) of sooty mould (A) and dieback (B) disease under Integrated Pest Management (IPM); Farmers Practice and Control of Kinnow during 2021-23; Error bar represent standard errors.

A low rate of AUDPC (Area Under Disease Progress Curve) of sooty mould was observed in T_1_-IPM (6921, 6355 & 5398) followed by T_2_-farmer practice (8443, 7891 & 6809) whereas, it was highest in T_3_-control (9992, 9246 & 9068) (Fig. 8A). Similarly, in case of dieback, low rate of AUDPC was also documented in T_1_-IPM (9268, 8911 & 8790) followed by T_2_-farmer practice (11106, 10698 & 10540) while it was higher in T_3_-control (13280, 11981 & 11834) (Fig. 8B). Overall, AUDPCs of sooty mould and dieback diseases were recorded highest during the year 2021 compared to 2022 & 2023.

### 3.6 Economics of IPM, farmer practice and in control

Economics of Kinnow mandarin in T_1_-IPM, T_2_-farmer practice and T_3_-control from 2021 to 2023 were computed considering various relevant parameters. It was evident that the average number of chemical sprays were reduced to 4.7 in T_1_-IPM compared to 11 in T_2_-farmer practice while no chemical spray of pesticides was given in T_3_-control. The total cost of plant protection alone in T_1_-IPM was less (Rs.19555/ha) compared to T_2_-farmer practice (Rs. 30909/ha). Similarly, the average total cost of cultivation in T_1_-IPM was lower (Rs.135708/ha) compared to T_2_-farmer practice (Rs.148125/ha) and T_3_-control (Rs.128250/ha), respectively. The highest fruit drop was noticed in T_3_-control (88.56 %) followed by T_2_-farmer practice (75.57 %) compared to T_1_-IPM (69.07 %) Highest average no. of fruits/tree, average fruit weight (g) and average fruit yield (kg/tree), respectively were also recorded in T_1_-IPM (357, 230 & 82) compared to T_2_-farmer practice (336.67, 204.33 & 68.67) and T_3_-control (194.33, 139.67 & 27). Lower average fruit yield (7.42 ton/ha) was recorded in T_3_-control and T_2_-farmer practice (18.88 ton/ha) compared to T_1_-IPM (22.46ton/ha). The highest fruit yield loss caused by psylla, whitefly, sooty mould and dieback was recorded in T_3_-control (66.96 %) compared to T_2_-farmer practice (15.94). Similarly, the maximum increase in fruit yield (ton/ha) over control and increase in fruit yield (ton/ha) over farmer practice, respectively (15.04 % & 31.19 %) was also found in T_1_-IPM. Similarly, the highest average net income was also noticed in T_1_-IPM (Rs.518436/ha) compared to T_2_-farmer practice (Rs.387366/ha) and T_3_-control (Rs. 94450/ha). Additional net profit due to increase in fruit yield over control, additional net profit due to increase in fruit yield over farmer practice, and additional profit due to saving no of spray were also higher (Rs.423986/ha, Rs.131070/ha & Rs.11354/ha) in T_1_-IPM compared to T_3_-control. Overall, the T_1_-IPM increased the net profit of Rs. 529790/ha compared to T_2_-farmer practice and T_3_-control. In terms of benefit-cost ratio, it was evident that a higher benefit-cost ratio was recorded in T_1_-IPM (4.34) compared to T_2_-farmer practice (3.16) and T_3_-control (1.73), respectively (Table 6).

**Table 6:** Economic analysis of pest management technologies in kinnow mandarin orchards during 2021–2023.

Particulars	2021			2022			2023			Average (2021-23)		
	IPM	FP	UC	IPM	FP	UC	IPM	FP	UC	IPM	FP	UC
Chemical spray (nos)	5	12	–	5	11	–	4	10	–	4.7	11	–
Cost of plant protection (Rs./ha)	20166	33102	–	20200	31125	–	18300	28500	–	19555	30909	–
Cost of cultivation (Rs./ha)	134625	146250	126000	135500	148125	128500	137000	150000	130250	135708	148125	128250
Fruit drop (%)	67.59	73.74	87.03	70.23	77.12	90	69.41	75.85	88.65	69.07	75.57	88.56
Average no.of fruits/tree	370	345	208	350	330	185	351	335	190	357	336.67	194.33
Average fruit weight (g)	228	206	145	223	200	135	238	207	139	230	204.33	139.67
Average fruit yield (kg/tree)	84	71	30	78	66	25	83	69	26	82	68.67	27.00
Fruit yield (ton/ha)	23.1	19.52	8.25	21.45	18.15	6.87	22.82	18.97	7.15	22.46	18.88	7.42
Fruit yield loss (%)	–	15.49	64.28	–	15.38	67.97	–	16.87	68.66	–	15.94	66.96
Increase in fruit yield (ton/ha) over control	14.85	11.27	–	14.58	11.28	–	15.67	11.82	–	15.03	11.46	–
Increase in fruit yield (ton/ha) over FP (%)	31.76	–	–	29.25	–	–	32.57	–	–	31.19	–	–
Gross income (Rs./ha)	693000	585600	247500	643500	544500	206100	684600	569100	214500	673700	566400	222700
Net income (Rs./ha)	538209	406248	121500	487800	365250	77600	529300	390600	84250	518436	387366	94450
Additional net profit due to increase in fruit yield (Rs./ha) over control	416709	284748	–	410200	287650	–	445050	306350	–	423986	292916.00	–
Additional net profit due to increase in fruit yield (Rs./ha) over FP	131961	–	–	122550	–	–	138700	–	–	131070	–	–
Additional profit due to savings of sprayings (Rs./ha)	12936	–	–	10925	–	–	10200	–	–	11354	–	–
Net profit due to IPM (Rs./ha)	551145	–	–	498725	–	–	539500	–	–	529790	–	–
Benefit cost ratio	4.47	3.26	1.96	4.13	3.03	1.6	4.41	3.18	1.64	4.34	3.16	1.73

IPM -Integrated pest management; FP - Farmers practice; UC -Untreated control; Input cost include labour charges, fertilizer cost, plant protection cost, spraying cost, fruit harvesting cost etc.; Total returns were calculated on average sale price @30/kg, (Govt. of India, 2023).

## 4 Discussion

The present investigation aimed to assess the impact of various IPM practice against psylla, whitefly, sooty mould and dieback pests of Kinnow mandarin. The IPM strategy adopted against psylla, whitefly, sooty mould, and dieback in Kinnow mandarin in present study was highly effective in terms of recording lower pests damage, higher fruit yield, lower plant protection cost, lower pesticides sprays in comparison to farmer practice and control. It has been observed earlier that psylla one of the most devastating insects, mostly appeared in March–April, July–August, and September–October months on mandarin new flush [9,12,[43], [44], [45]]. It has been found previously that initially psylla appeared in small patches in the orchard but subsequently spread to all Kinnow mandarin orchards [9,46] and most importantly, it transmits the greening disease from disease to healthy plant [9,47,48]. In present study, installation of yellow sticky traps, use of NSKE 5 % on young leaves, intercropping with onion, garlic, and chickpea, and transplanting of marigold (5:1) did not only lowered the population of psylla but also enhanced the population of natural enemies. We also observed that when the psylla was exceeded ETL (6 no. of nymphs and adults/10 cm shoot) the spray of label claim imidacloprid 17.8SL @ 200 ml in 500l water proved very effective in reducing its population. Previous studies [[49], [50], [51], [52]] revealed that the application of IPM practices *viz*; use of biological control, use of green pesticides, cultural control, clean cultivation, monitoring of pests in the field, and intercropping of onion, garlic, chickpea with the main crop were found very effective in reducing the pest populations and provided optimum conditions for the multiplication and augmentation of natural enemies. Likewise, yellow sticky traps have been found to be useful in monitoring and studying population dynamics of psylla earlier [53,54]. Furthermore, highest percentage reduction of the psylla population was recorded in IPM (74.80 %) compared to farmer practice (40.50 %) after analysis of different IPM modules including NSKE5% [16] which corroborates our study.

In our study, presence of whitefly was observed on young both nymphs and adults sucked the sap from new flush and earlier whitefly has been found infesting citrus across growing tracts of India [47]. In the present investigation, installation of a yellow sticky traps has important monitoring tool for small insects especially aphids, whitefly [55]. Previously, it has also been found that yellow sticky traps were predominately used for determined the first emergence and population peak of whitefly enabled to take required management action in time. In our study, installation of yellow sticky traps @ 20/ha curbed the whitefly population below ETL (4–10 nymphs and adults/leaf) when use in combination with other IPM strategy. Earlier, installation of yellow sticky traps @ 10/ha reduced the population of soft-bodied insects in IPM compared to farmer practice [16] which validates our work. Similarly, good agriculture practices as adopted in IPM also have an important role in reducing populations of mites, scales, aphids, and whiteflies as reported previously [56]. In our findings, spray of NSKE @ 5 % and imidacloprid 17.8SL @ 200 ml in 500-L water effectively controlled psylla and whitefly which are similar to reports of previous researchers [52,57]. In a similar way, soil and foliar use of thiamethoxam at 0.008 % and foliar spray of imidacloprid at 0.009 % were found quite effective in managing the psylla population [58] which corroborates our study. We observed that spraying of systemic insecticides on the lower side of leaves during peak population checked the pest attack effectively. Earlier, imidacloprid and other systemic insecticides proved most effective against sucking insects than the contact insecticides [[59], [60], [61]] which are also in agreement with our results. Besides, insecticide deposition on leaf by spray also reduced the feeding and oviposition of psylla [[62], [63], [64]]. Foliar spray of imidacloprid 200 SL @ 0.25 ml followed by thiamethoxam 20 WG @ 0.1 g or acetamiprid 20 SP @ 0.1 g/l water followed by neem oil @ 1 % has earlier been found very effective against whitefly which also supported our study [9]. Moreover, psylla and other sucking insects create panic in Kinnow mandarin growers, forcing them to resort to over use of pesticides in a desperate attempt to stop the threat.

IPM strategy also proved effective against sooty mould and dieback in Kinnow mandarin orchards. Psylla and whitefly excrete honeydew which attracts the sooty mould and other honeydew-loving fungus [65] and as reported earlier under high psylla and whitefly populations, the incidence of sooty mould becomes high that reduces market value, quality, and attractiveness of fruits [66]. In our investigation, severity of sooty mould was high which lowered down by 1 % starch spray. Definitely, timely pruning of infected branches and their immediate destruction followed copper oxychloride (3 g/l) spray prevented further spread of dieback disease. In orchards, the symptoms of dieback were more pronounced during the June and July months due to the rainy season. It has been reported that sometimes, Kinnow mandarin plants wilted and died with the holding of fruits due to high severity and farmer faced huge monetary losses [67] which was managed by timely pruning and destruction of dead twigs and a spray of copper oxychloride @ 0.3 %. Earlier dieback was managed by using two sprays of copper oxychloride @ 0.25 % during July and August and the treatment also recorded lower fruit drop (84.75 %) compared to control (90.18 %) [22] which also supported our study. Furthermore, different fungicides including copper oxychloride could effectively manage the dieback disease [68]. In our study, incidence and severity of dieback was always in higher side which supported by Ref. [69]. Natural enemies also play a potent role in managing psylla and whitefly. In present study, highest populations of natural enemies were recorded in IPM and control treatment. Likewise, adoption of ecological engineering (marigold and sunflower, etc.) and growing of onion/garlic/chickpea between the row of Kinnow mandarin tree played a significant role along with other IPM practices for increasing the populations of natural enemies and further in reducing the damage of psylla and whitefly. These IPM practices not only controlled the populations of psylla and whitefly but also increased the extra income of farmers, conserved the soil moisture, and suppressed the weeds. In present study, it was also noticed that populations of natural enemies were less in farmer practice due to injudicious sprays of pesticides. Farmers applied 8–12 sprays of different pesticides including insecticides and fungicides which were higher than recommended dose. Fruit drops are mostly caused by complex pathogen infections, types of fertilizer and growth regulators used. Earlier, it has been observed that, fruits mostly drop in the months of November, December, and January as the fruit near the maturity [71] and have major bottleneck for the citrus grower [70]. Remarkably, in our study, spray of aureofungin/bavistin +2,4-D significantly reduced the fruit drop in Kinnow mandarin and minimum fruit drop (69.07 %) was noticed in IPM compared to farmer practice which corroborates by Ref. [72], who used fungicides in combination with 2,4-D @ 20 ppm and KNO_3_ @ 1 % were found to significantly reduce fruit drop compared to control. It was also observed that fungicides, small concentrations of 2,4-D and combined application of fungicide with 2, 4-D were responsible for maximum fruit retention in Kinnow mandarin [71,73,74] which are agreement with our investigation. The economic effectiveness of various pest management modules showed an increased marketable yield over control. In present study, IPM registered a reduced number of chemical sprays (4.7), lowest cost of plant protection, higher fruit yield, higher net income and maximum benefit-cost ratio (4.34) compared to farmer practice and control that are similar to Ref. [16], who earlier recorded a higher yield of 131.78 kg/tree with a high cost: benefit ratio of 1: 3.95 in IPM compared to 97.67 kg/tree with a cost: benefit ratio of 1:1.95 in farmer's practice. Additionally, various IPM modules in farmer's participatory mode were evaluated and found to be higher yield and benefit-cost ratio compared to farmer practice [75] that are in close agreement with our finding.

Besides, farmers were advised and equipped with improved knowledge and skills which helped them with the use of appropriate IPM practices in Kinnow mandarin. Demonstrations of NSKE 5 %, pruning, farmers' gosthi, farmers’ field schools (FFS), and Kinnow mandarin day were also organized at regular intervals to create awareness among the farmers about identification of pest, natural enemies, nature of pest damage, and application of IPM components. IPM practices hold a key role in reducing pest damage without interfering with ecological balance, reduce pesticide residues, enhancing ecosystem services, increasing the income of farmers, and strengthening farmer knowledge.

## 5 Conclusion

To conclude, the present study highlights that IPM strategy (cultural, mechanical, and chemical) were found economically viable options for the management of psylla, whitefly, sooty mould, and dieback in Kinnow mandarin. The validated IPM strategies were found very effective and proved efficient in tackling the menace of psylla, whitefly, sooty mould, and dieback in comparison to the farmer practice. As a result of a reduction in infestation of psylla, whitefly and severity of sooty mould and dieback due to IPM strategy despite fewer pesticides use, farmers profited by the production of higher good quality fruit yield, higher market price and benefit-cost ratio for Kinnow mandarin. The validated IPM strategy *viz*; installation of yellow sticky traps @ 20/ha followed by spray of neem seed kernel extract (NSKE) @ 5 % and imidacloprid 17.8SL @ 0.3 % against psylla and whitefly and 1 % starch spray and dipping infected fruits in a bleaching solution @ 1 % against sooty mould and pruning followed by spray of copper oxychloride @ 0.3 % on dieback are thus recommended as key pest management strategies for sustainable production of Kinnow mandarin.

## CRediT authorship contribution statement

**Prabhu Narayan Meena:** Writing – original draft, Methodology, Investigation, Data curation, Conceptualization. **D. Raghavendra:** Resources, Methodology, Formal analysis. **Satyendra Singh:** Validation, Investigation, Formal analysis, Data curation, Conceptualization. **Narendra Kumar:** Validation, Methodology, Formal analysis, Data curation. **Mukesh Kumar Khokhar:** Validation, Resources, Investigation, Formal analysis, Data curation. **Subhash Chander:** Writing – review & editing, Supervision, Resources, Project administration, Investigation. **Milan Kumar Lal:** Writing – review & editing, Supervision, Methodology, Formal analysis, Data curation. **Rahul Kumar Tiwari:** Writing – review & editing, Validation, Software, Methodology, Funding acquisition, Formal analysis. **Ravinder Kumar:** Writing – review & editing, Validation, Supervision, Resources, Methodology, Funding acquisition, Formal analysis.

## Ethics approval and consent to participate

Not applicable.

## Consent for publication

Yes.

## Availability of data and materials

The datasets generated during and/or analysed during the current study are available from the corresponding author on reasonable request.

## Funding

Not available.

**Table:** List of abbreviations

IPM	Integrated Pest Management
SMW	Standard Meteorological Week
AUDPC	Area Under Diseases Progress Curve
KVK	Krishi Vigyan Kendra
RBD	Randomized Block Design
FYM	Farm Yard Manure
ETL	Economic Threshold Level
PSI	Percentage Severity Index
FP	Farmer Practice

## Declaration of competing interest

The authors declare the following financial interests/personal relationships which may be considered as potential competing interests:Dr. Ravinder Kumar (corresponding author) is associate editor of Heliyon Journal-Agriculture Section. If there are other authors, they declare that they have no known competing financial interests or personal relationships that could have appeared to influence the work reported in this paper.
